# Health Tract, No. 194

**Published:** 1865

**Authors:** 


HEALTH TRACT, No. 194.

### MEMORIES.

"ALL men think all men mortal but themselves." Every man goes on the presumption that he at least, whatever may be the fate of the friends of his own age, will live to become an old man; and taking it for granted that such will prove to be the fact, the very commonest capacity ought to feel the importance and see the wisdom of so living, that when that old age does come, it shall be one of physical comfort and mental repose; that, in short, it shall be an old age, genial, joyous, and gleesome. Regular, temperate, and industrious habits from youth through mature years will, with the utmost certainty, give health and vigor to gray hairs, without pain or sickness or premature wasting. But at three-score and ten, the step may be still firm, the eye bright, the intellect vigorous, digestion good, and all the tastes, appetites, propensities, and appreciations still keen and enjoyable; but in that bright eye there is no merry twinkle; in that yet ruddy face there is no index of a genial heart within; the voice, still strong, does not express itself in sympathetic benevolences; and the hands are all unused to deeds of love and kindness to the unfortunate and the poor; and, in addition, there is a hardness of sentiment and manner and conduct, which prove, beyond contradiction, that within that bosom there is not one ray of pure joy, not one thrill of unalloyed delight. And why? All the energies, from youth to age, had been expended in securing food for the body, as if human existence were only to eat and drink and die. No provision had been made for the aliment of the mind and heart, as if they needed nothing but to gloat over hoarded gold. But in amassing that treasure, injustices were done; wrongs had been perpetrated; deceits had been practiced; advantages had been taken of brother-strugglers in the race for life. It had altogether been forgotten that the heart of age fed best, and flourished green and sunshiny, on the sweet memories of good deeds done, of kind acts performed by favoring the poor, aiding the weak, assisting the unfortunate, helping up the fallen, encouraging the despondent, counseling the ignorant, steadying the wayward, warning the reckless, and being a brother, forbearing, loving, considerate, toward all, as the representatives of a common Father in heaven and the Saviour of man, and failing thus to do "to one of these little ones," they had shown themselves unwilling to do it to the Infinite, who made, preserved, and redeemed them; thus, when age comes, the heart has nothing to live upon, the busy memories, sharp-pricking, go back to clouds instead of sunshine, and the man feels, as did that great but perverted intellect,

"The flowers, the fruits are gone;
The worm, the canker, and the grief
Are mine alone!"

But let a man feel that the truest way of living for himself is to live for others; that the best way to serve his Maker is to "make it his meat and his drink," his highest aim, to benefit and bless mankind habitually, by such acts of kindness and charity as it is in his power to perform, consistent with the other duties of life; then the earthly pilgrimage will have a very different ending; for as he enters upon immortal scenes, he exclaims, not in the despairing language of Lord Byron, but in the exultant expressions of one quite as highly born as the English nobleman, of talents quite as commanding, and, in the learning of his time, very far superior: "The time of my departure is at hand; I have fought a good fight, I have finished my course, I have kept the faith; henceforth there is laid up for me a crown of righteousness, which the Lord, the righteous Judge, shall give me at that day; and not to me only, but unto all them also that love his appearing."

